# Psychotropic drug utilization pattern among patients with schizophrenia

**DOI:** 10.4103/0019-5545.43066

**Published:** 2005

**Authors:** Shakti Bala Dutta, D.C. Dhasmana, Reena Bhardwaj

**Affiliations:** * Assistant Professor, Department of Pharmacology, Himalayan Institute of Medical Sciences, Swami Rama Nagar, Doiwala, Dehradun 248140, Uttaranchal; ** Professor and Head, Department of Pharmacology, Himalayan Institute of Medical Sciences, Swami Rama Nagar, Doiwala, Dehradun 248140, Uttaranchal; *** Postgraduate Student, Department of Pharmacology, Himalayan Institute of Medical Sciences, Swami Rama Nagar, Doiwala, Dehradun 248140, Uttaranchal

Sir,

Schizophrenia is a chronic, debilitating, major psychiatric disorder that may require life-long treatment. Treatment of such patients at an early stage may improve the rates of morbidity and mortality, as well as productivity. Till date, pharmacotherapy remains the cornerstone of treatment in such patients. The serendipitous discovery of chlorpromazine and lithium in the 1950s brought about a revolution in the field of psychopharmacology. Although highly efficacious, the side-effects^1^ of these older conventional drugs were distressing, leading to an active search for better agents. The advent of newer antipsychotic agents such as olanzapine, risperidone and quetiapine has led to major changes in the drug usage pattern.

Presently, a number of antipsychotic agents are available and this, at times, makes it difficult to select a particular antipsychotic drug on a rational basis and not empirically. These agents are increasingly being used for a variety of indications. Keeping these factors in mind, a prospective study was conducted by the Department of Pharmacology in the Psychiatry OPD of the Himalayan Institute Hospital—a tertiary-care teaching hospital in Uttaranchal—during 2002–2003. The aim of the study was to analyse the prescribing pattern of drugs in stable patients with chronic schizophrenia, and to correlate the availability of newer medicines in the market and their usage on current psychiatric practice. One hundred and eighteen adult stable patients with schizophrenia, diagnosed according to the DSM-IV criteria,^2^ were included in the study. Individual data were collected in a predesigned format and analysed on parameters such as the distribution of subsets of the disease, demographic profile and psycho-tropic drug use pattern.

Results showed the following distribution of different types of schizophrenia: paranoid 51.7% (61), disorganized 5.9% (7), catatonic 10.2% (12), undifferentiated 3.4% (4), residual 11.8% (14), episodic, i.e. interepisodic residual symptoms with prominent negative symptoms 10.1% (12) and episodic with no interepisodic symptoms or continuous with prominent negative symptoms 6.7% (8). There were 64 males (54.23%) and 54 females (45.76%); 68 patients (57.62%) were <30 years of age and 50 (42.37%) were >30 years. As regards the socioeconomic status, 45 patients belonged to the upper income group, 62 to the middle income group and 11 to the low income group. Of the total patients, 28 were professionals, 51 non-professionals, 23 students and 16 labourers or farmers.

Table 1 gives the drug use pattern of patients with schizo-phrenia. A total of 338 drugs were prescribed, out of which 316 were given orally and 22 parenterally as depot formulations. The different antipsychotics used were: olanzapine 44.4%, haloperidol 28.5%, risperidone 9.5%, quetiapine 6.3%, aripiprazole 5.2%, ziprasidone 4.2% and thioridazine 2.6%. Of the total 189 antipsychotic medications used orally, 58 patients were prescribed only one antipsychotic drug, 49 were prescribed two and 11 were given more than two antipsychotics. Of the anticholinergics, trihexyphenidyl was prescribed for 64 patients.

**Table 1:** Drug use pattern of patients with schizophrenia in the Psychiatry OPD

Total number of prescriptions	118
Total number of drugs used	338
Average number of drugs per prescription	2.8
Number of fixed-dose combinations	8
Types of psychotropic drugs used	
Antipsychotics	189 (55.9%)
Antidepressants	23 (6.8%)
Antimanics	12 (3.5%)
Anxiolytics	18 (5.3%)
Anticholinergics	64 (18.9%)
Miscellaneous	32 (9.4%)

A trend towards multidrug therapy was observed. Also evident was a shift towards the use of newer atypical anti-psychotic agents. Concomitant anticholinergics were used only in about half of the patients, reflecting their reduced usage with the increasing use of newer atypical antipsychotics. Fixed-dose combinations were also prescribed occasionally. Other psychotropic medications prescribed as adjunctive treatment were antidepressants (venlafaxine and fluoxetine), anxiolytics (clonazepam) and antimanic agents (sodium valproate).

The treatment pattern observed correlates with the changing trends in the treatment of schizophrenia the world over. In recent years, newer atypical antipsychotic agents such as olanzapine, risperidone, quetiapine and ziprasidone have provided a better control of symptoms and have fewer adverse effects, especially extrapyramidal ones, in contrast with the older drugs. The newer atypical antipsychotics have also proved to be better in improving the negative symptoms, cognitive dysfunction and are efficacious in neuroleptic-resistant schizophrenia.^3^,^4^ With the recent introduction of the third-generation antipsychotic agent aripiprazole, which acts by stabilizing the serotonin–dopamine system, further improvement in the management of patients with schizophrenia is envisaged.
